# Vine Tea (*Ampelopsis grossedentata*) Extract Mitigates High-Salt-Diet-Induced Hypertension by Remodeling the Gut Microbiota–Metabolite Axis in Mice

**DOI:** 10.3390/ijms27020709

**Published:** 2026-01-10

**Authors:** Yuxuan Gu, Qiling Li, Lu Cao, Huabing Yang

**Affiliations:** 1 School of Basic Medical Sciences, Hubei University of Chinese Medicine, Wuhan 430065, China; guux0616@163.com (Y.G.); 18723760383@163.com (Q.L.); 2 Hubei Shizhen Laboratory, Wuhan 430065, China

**Keywords:** vine tea, hypertension, high-salt diet, gut microbiota, metabolomics, cardiorenal injury

## Abstract

Hypertension is a major global health challenge, with excessive dietary salt intake recognized as a key environmental factor contributing to its pathogenesis. However, safe and effective dietary interventions for salt-sensitive hypertension remain limited. Vine tea (*Ampelopsis grossedentata*), a traditional herbal tea widely consumed for centuries in southern China, has been reported to exhibit antioxidant, anti-inflammatory, and hepatoprotective activities, yet its antihypertensive efficacy and underlying mechanisms remain unclear. In this study, the chemical profile of vine tea aqueous extract (VTE) was characterized by UPLC–Q–TOF–MS, identifying dihydromyricetin, isoquercitrin, and myricetin as the predominant flavonoids. The protective effects of VTE were evaluated in C57BL/6J mice with high-salt-diet (HSD)-induced hypertension. VTE treatment significantly lowered systolic blood pressure and ameliorated cardiac and renal injury, accompanied by reduced inflammation, fibrosis, and cardiac stress-related gene expression. Gut microbiota analysis using 16S rRNA gene sequencing revealed that VTE restored microbial richness and diversity, enriching short-chain fatty acid-producing taxa while suppressing pathogenic *Desulfovibrio* and *Ruminococcus torques*. Untargeted plasma metabolomic profiling based on UPLC–Q–TOF–MS further showed that VTE normalized tryptophan, bile acid, and glycerophospholipid metabolism, decreasing the uremic toxin indoxyl sulfate while increasing tauroursodeoxycholic acid. Notably, these protective effects were abolished under antibiotic-induced microbiota depletion, confirming that VTE acts through a gut microbiota-dependent mechanism. Collectively, VTE mitigates salt-induced hypertension and cardiorenal injury by remodeling the gut microbiota–metabolite axis, supporting its potential as a natural dietary intervention for managing hypertension.

## 1. Introduction

Hypertension is one of the most prevalent chronic diseases worldwide and remains the leading risk factor for cardiovascular morbidity and mortality [1,2,3]. According to the Global Burden of Disease Study, over one billion people are affected, with hypertension contributing to more than 10% of global deaths each year [4]. Excess dietary sodium intake is a major environmental factor driving hypertension and its complications, including cardiac hypertrophy, heart failure, and chronic kidney disease [5,6]. A high-salt diet (HSD) not only elevates blood pressure but also aggravates target organ injury by promoting vascular dysfunction, immune activation, and metabolic disturbance [7,8].

Although several classes of antihypertensive agents—such as angiotensin-converting enzyme inhibitors (ACEIs) [9,10], angiotensin II receptor blockers (ARBs) [11,12], calcium channel blockers (CCBs) [13], β-adrenergic blockers [14], and diuretics [15]—are clinically available, their long-term efficacy and safety remain limited. These drugs primarily lower blood pressure without reversing pre-existing organ damage, and their use is often accompanied by side effects including cough [16], hyperkalemia [17], or peripheral edema [18]. Moreover, these pharmacological agents typically target single pathways, such as the renin–angiotensin or calcium signaling systems, and have limited influence on the metabolic and microbial disturbances that underlie salt-sensitive hypertension [19,20,21]. Therefore, there is an increasing interest in identifying safer, long-term, and multi-targeted strategies that not only reduce blood pressure but also protect against hypertension-induced organ damage.

Recent evidence highlights the gut microbiota as a critical regulator of blood pressure homeostasis [22,23]. High-salt intake disrupts microbial homeostasis by reducing beneficial commensals and enriching pro-inflammatory taxa, contributing to the onset and progression of hypertension [24]. Mechanistically, gut microbes regulate host physiology through bioactive metabolites such as short-chain fatty acids (SCFAs), bile acids, and tryptophan derivatives [25]. For instance, SCFAs enhance vascular and immune homeostasis [26], whereas elevated trimethylamine N-oxide promotes vascular inflammation and oxidative stress [27]. Given this central role of the gut microbiota–metabolite axis in salt-sensitive hypertension, interventions capable of restoring microbial and metabolic balance have attracted growing interest. In particular, plant-derived polyphenols and flavonoids have been shown to beneficially modulate this axis, enhancing antioxidant defense, reducing inflammation, and improving intestinal integrity in experimental models [28,29,30,31]. These findings underscore the potential of natural dietary compounds as multi-target modulators of gut microbiota and host metabolism, offering a safer and more sustainable strategy for hypertension prevention and management.

Vine tea (*Ampelopsis grossedentata*), a traditional medicinal and edible plant widely consumed in southern China [32], has been used for more than a thousand years to relieve inflammation, clear heat, lower lipids, and reduce blood pressure [33]. Modern pharmacological studies have revealed that vine tea is rich in bioactive compounds, such as flavonoids, phenols, and steroids, which exhibit anti-inflammatory, antioxidant, hepatoprotective, and lipid-lowering properties [34]. In contrast to conventional antihypertensive drugs that act on single molecular pathways such as the renin–angiotensin system, vine tea comprises multiple constituents capable of influencing interconnected metabolic and microbial processes [34]. This inherent multi-target potential, combined with its established safety as a dietary substance, suggests that VTE may provide a systems-level therapeutic approach for managing hypertension and related metabolic disorders. However, despite these pharmacological insights, the antihypertensive efficacy of VTE and the underlying mechanisms, the antihypertensive potential and underlying mechanisms of vine tea, particularly those involving gut microbiota and host metabolism, remain poorly understood.

In this study, we hypothesized that VTE could mitigate HSD-induced hypertension through modulation of the gut microbiota–metabolite axis. To test this hypothesis, we performed integrating physiological, histopathological, 16S rDNA sequencing, and untargeted metabolomic analyses in conventional and microbiota-depleted mice. This study provides new mechanistic insight into the microbiota-dependent protective effects of VTE and supports its potential as a natural dietary intervention for salt-sensitive hypertension.

## 2. Results

### 2.1. Phytochemical Composition of VTE

**Figure 1:**
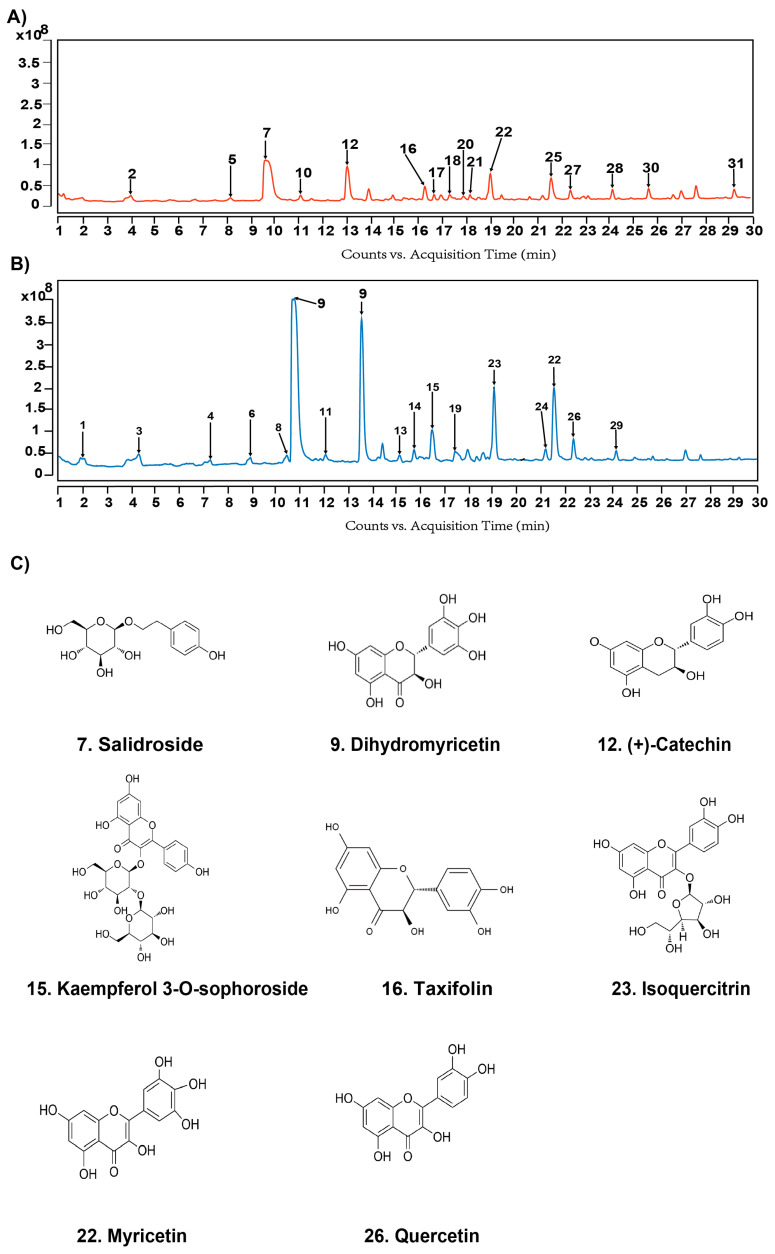
Chemical profiling of Vine Tea Extract (VTE) by UPLC-Q-TOF-MS. (**A**) Base peak chromatogram (BPC) of VTE in positive ion mode. (**B**) BPC of VTE in negative ion mode. (**C**) Chemical structures of the major constituents identified in VTE.

A total of 31 compounds were identified in VTE by UPLC-Q-TOF-MS under positive and negative ion modes (Figure 1A,B). Detailed compound information is provided in Table A1. Most constituents were flavonoids, including four flavan-3-ols (peaks 2, 5, 11, and 12), two dihydroflavonols (peaks 9 and 16), and a range of flavonol derivatives (peaks 3, 4, 13, 14, 15, 18, 19, 22, 23, 24, 26, 27, and 30). Additional polyphenols were also detected, including a phenolic acid (peak 1), a stilbene (peak 6), and a phenylethanoid glycoside (peak 7). Other structural classes included one lignan/phenylpropanoid derivative (peak 28), one coumarin derivative (peak 21), one anthraquinone glycoside (peak 29), and one terpenoid glycoside (peak 31). Among these, dihydromyricetin (peak 9) was the most abundant compound, followed by isoquercitrin (peak 23), myricetin (peak 22), and quercetin (peak 26), Salidroside (peak 7), (+)-Catechin (peak 12), Kaempferol 3-O-sophoroside (peak 15), Taxifolin (peak 16) (Figure 1A–C).

### 2.2. VTE Attenuates HSD-Induced Hypertension and Related Phenotypes

**Figure 2:**
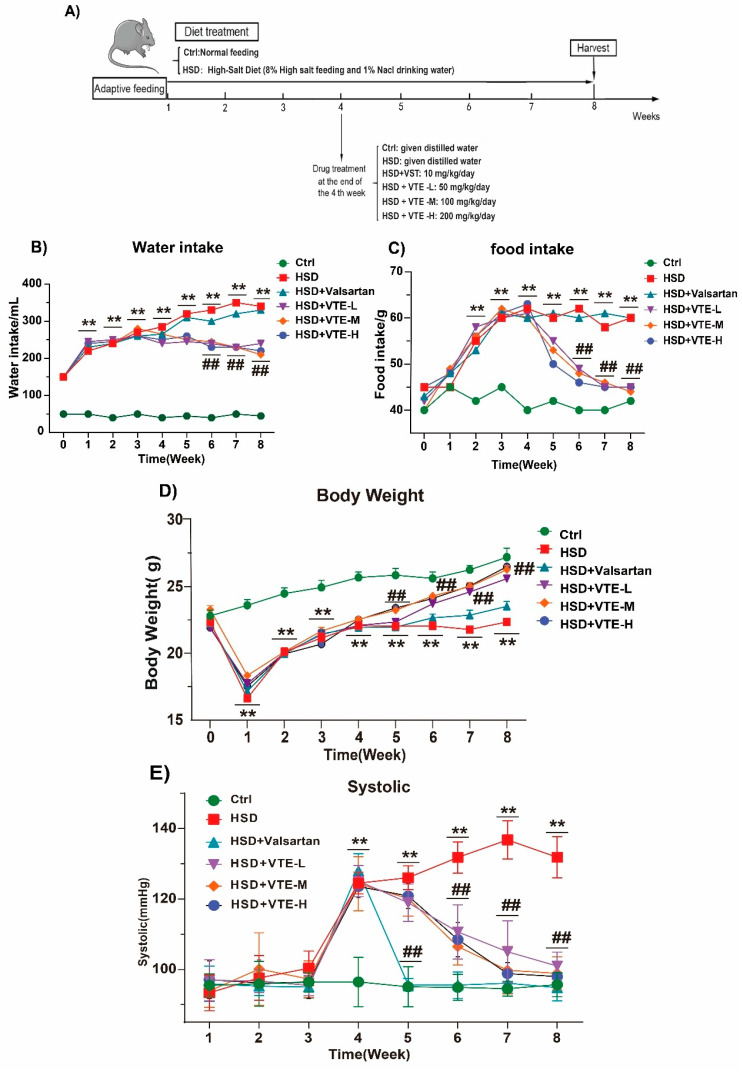
Effects of VTE on physiological parameters in HSD-induced hypertensive mice. (**A**) Schematic diagram of the experimental timeline. (**B**) Daily water intake. (**C**) Daily food intake. (**D**) Body weight changes. (**E**) Systolic blood pressure. Data are presented as mean ± SEM (*n* = 10). ** *p* < 0.01 vs. Ctrl group; ^##^ *p* < 0.01 vs. HSD group.

A hypertension model was established by feeding mice an 8% high-salt diet (HSD) combined with 1% NaCl drinking water (Figure 2A). After four weeks, systolic blood pressure (SBP) rose sharply, confirming successful induct ion of hypertension (Figure 2E). Animals then received daily oral administration of either Valsartan (10 mg/kg) or VTE at low, medium, or high doses (50, 100, or 200 mg/kg). Compared to the Ctrl group, HSD-fed mice exhibited increased food and water intake, as well as significant body weight loss (Figure 2B,C). After two weeks of intervention, VTE treatment significantly ameliorated these alterations: it attenuated the elevated SBP (Figure 2E), reduced food and water intake, and partially reversed body weight loss (Figure 2B–D). In contrast, Valsartan effectively lowered SBP (Figure 2E) but did not significantly alter the HSD-induced increases in food/water intake or body weight loss (Figure 2B–D).

### 2.3. VTE Protects Against HSD-Induced Cardiac and Renal Injury

**Figure 3:**
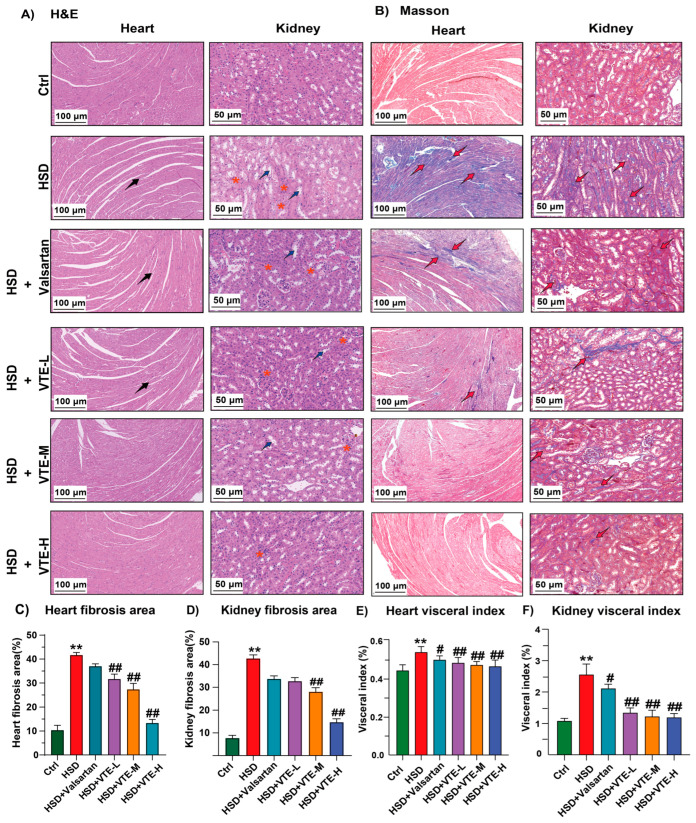
VTE ameliorates cardiac and renal injury in HSD-induced hypertensive mice. (**A**) Heart and kidney tissues were analyzed histomorphologically using hematoxylin and eosin (H&E) staining (heart scale bar = 100 μm, microscopic image magnification ×200; kidney scale bar = 50 μm, microscopic image magnification ×400). Black arrows indicate sites of myocardial fiber rupture. Blue arrows highlight tubular epithelial cytoplasmic vacuolization and detachment in the kidney. Red asterisks denote areas of inflammatory cell infiltration. (**B**) Heart and kidney tissues were analyzed histomorphologically using Masson’s trichrome staining (heart scale bar = 100 μm, microscopic image magnification ×200; kidney scale bar = 50 μm, microscopic image magnification ×400). Red arrow indicates the area of fibrosis. (**C**) Quantitative analysis of cardiac fibrosis area from Masson’s trichrome staining. (**D**) Quantitative analysis of renal fibrosis area from Masson’s trichrome staining. (**E**) Heart visceral index. (**F**) Kidney visceral index. Data are presented as mean ± SEM. ** *p* < 0.01 vs. Ctrl group; ^#^ *p* < 0.05, ^##^ *p* < 0.01 vs. HSD group.

To assess tissue-level protection, cardiac and renal morphology, fibrosis, and organ indices were examined. H&E staining revealed disorganized cardiomyocytes, fiber fractures, and intercellular widening in HSD-fed mice. In the kidneys, HSD induced tubular epithelial atrophy, glomerular structural disruption, and inflammatory infiltration. VTE administration markedly improved these pathological features in a dose-dependent manner, while Valsartan exhibited only a slight but non-significant tendency toward amelioration (Figure 3A). Masson staining demonstrated extensive fibrosis in both heart and kidney tissues following HSD feeding. VTE significantly reduced fibrotic areas, while Valsartan did not yield notable improvement (Figure 3B). Quantitative analysis confirmed substantial decreases in fibrosis ratios in VTE-treated mice compared with the HSD group (Figure 3C,D). Consistently, cardiac and renal organ indices were elevated in the HSD group but were markedly reduced by VTE treatment, with a stronger effect than Valsartan (Figure 3E,F).

**Figure 4:**
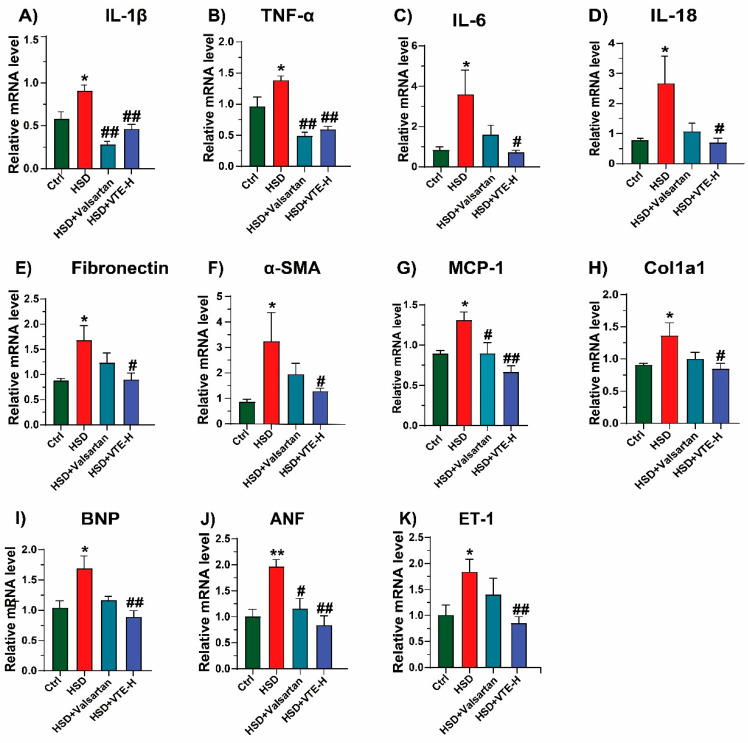
VTE attenuates cardiac inflammation, fibrosis, and dysfunction in HSD-induced hypertensive mice. Relative mRNA expression levels in cardiac tissues of (**A**) *IL-1β*, (**B**) *TNF-α*, (**C**) *IL-6*, (**D**) *IL-18*, (**E**) *Fibronectin*, (**F**) *α-SMA*, (**G**) *MCP-1*, (**H**) *Col1A1*, (**I**) *BNP*, (**J**) *ANF*, and (**K**) *ET-1*. Data are presented as mean ± SEM (*n* = 8). * *p* < 0.05, ** *p* < 0.01 vs. Ctrl group; ^#^ *p* < 0.05, **^##^** *p* < 0.01 vs. HSD group.

We next focused on the heart, as cardiac remodeling represents a major manifestation of hypertension-induced target organ damage. At the molecular level, HSD markedly upregulated inflammatory cytokines (*IL-1β*, *TNF-α*, *IL-6*, *IL-18*), fibrosis-related genes *(Fibronectin*, *α-SMA*, *Col1A1*), and cardiac stress markers (*BNP*, *ANF*, *MCP-1*, *ET-1*) compared with controls. High-dose VTE treatment (VTE-H) significantly suppressed the expression of all these genes, indicating broad anti-inflammatory and anti-fibrotic effects. In contrast, Valsartan only reduced HSD-induced elevations of *IL-1β*, *TNF-α*, *MCP-1*, and *ANF*, showing a limited molecular response (Figure 4A–K).

Taken together, these results indicate that VTE effectively mitigates HSD-induced cardiac and renal impairment in mice, showing greater improvement than Valsartan.

### 2.4. VTE Reshapes Gut Microbiota Composition in HSD-Fed Mice

**Figure 5:**
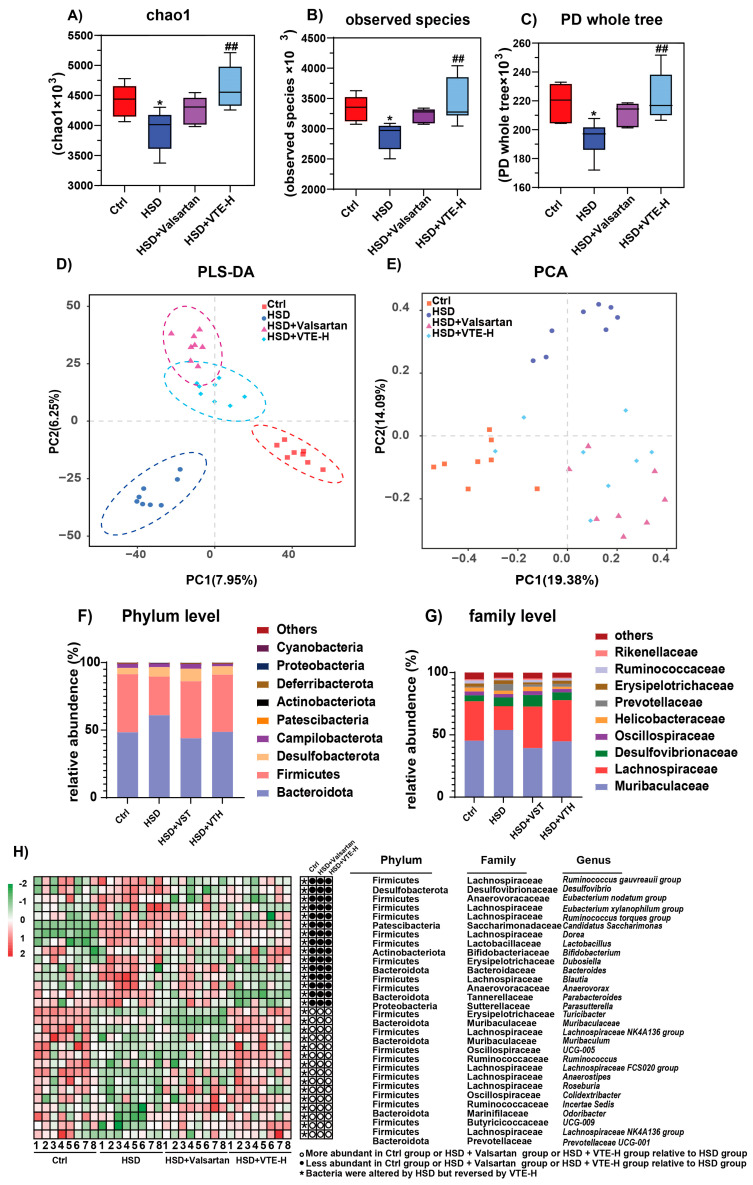
VTE alters the composition and structure of the gut microbiota in HSD-induced hypertensive mice. (**A**–**C**) Alpha diversity indices assessed by the (**A**) Chao1 index, (**B**) Observed species, and (**C**) Phylogenetic Diversity (PD) whole tree. (**D**) Partial least squares-discriminant analysis (PLS-DA). (**E**) Principal component analysis (PCA) of microbial communities. (**F**) Microbial composition at the phylum level. (**G**) Microbial composition at the family level. (**H**) Heatmap depicting relative abundance of gut microbiota at the genus level. Data are presented as mean ± SEM (*n* = 8). * *p* < 0.05 vs. Ctrl group; **^##^** *p* < 0.01 vs. HSD group.

Given the emerging link between gut microbiota and hypertension, 16S rDNA sequencing was conducted on fecal samples from Ctrl, HSD, HSD + Valsartan, and HSD + VTE-H groups. Alpha diversity analysis revealed that HSD significantly decreased microbial richness and diversity, as indicated by lower Chao1, Observed species, and PD whole tree indices. VTE-H administration significantly restored these metrics (Figure 5A–C), indicating a recovery of overall microbial diversity. Beta diversity analysis using principal component analysis (PCA) and Orthogonal projections to latent structures-discriminant analysis (OPLS-DA) revealed distinct microbial clustering among groups, with the VTE-H group showing a community profile closer to that of controls (Figure 5D,E).

At the phylum level, HSD increased *Bacteroidota* and *Desulfobacterota* while decreasing Firmicutes. VTE-H normalized these alterations, whereas Valsartan produced similar effects but failed to reduce the elevated abundance of *Desulfobacterota* (Figure 5F). At the family level, HSD reduced *Lachnospiraceae* and *Ruminococcaceae*—major SCFA producers [35]—but increased pathogenic *Desulfovibrionaceae* and slightly elevated *Muribaculaceae*. Both interventions restored beneficial taxa, though VTE-H had stronger effects (Figure 5G). Genus-level analysis showed that HSD enriched harmful bacteria such as *Desulfovibrio* and *Ruminococcus*, while depleting beneficial genera including *Bifidobacterium*, *Lactobacillus*, *Roseburia*, *Eubacterium xylanophilum group*, and *Lachnospiraceae*. VTE-H reversed these alterations more effectively than Valsartan (Figure 5H).

**Figure 6:**
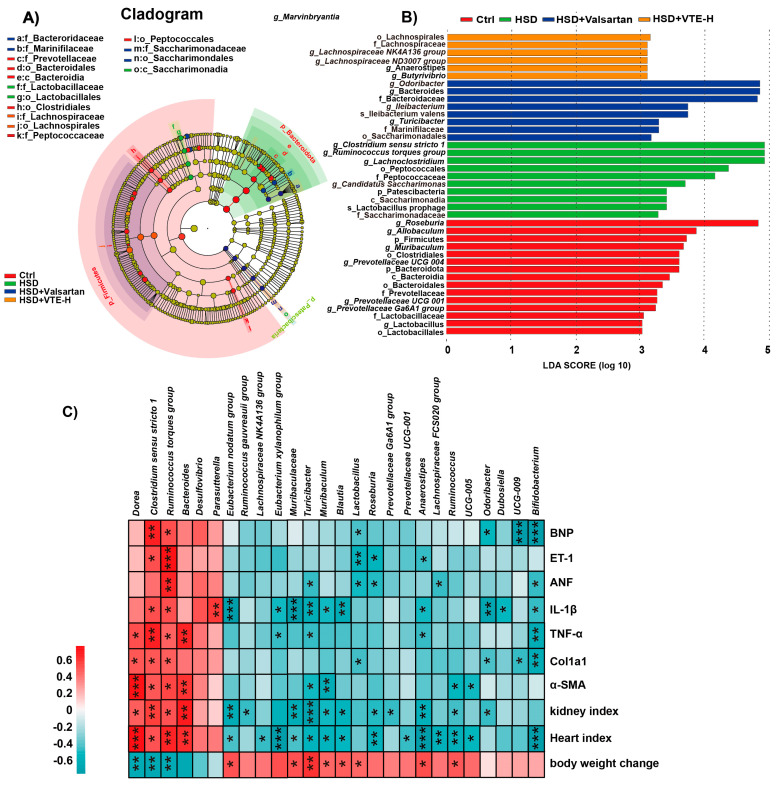
Correlation and taxonomic characterization of gut microbiota in response to HSD and VTE treatment. (**A**) Phylogenetic Cladogram (**B**) Linear discriminant analysis (LDA) scores of significantly enriched taxa. An LDA score > 3.0 indicates a higher abundance in the corresponding group. (**C**) Spearman’s correlation analysis between gut microbial genera at the genus level and physiological parameters (including microbial taxa, fibrosis levels, and inflammatory markers). The color gradient represents the correlation coefficient, from blue (negative correlation) to orange (positive correlation). Data are presented as mean ± SEM (*n* = 8). * *p* < 0.05, ** *p* < 0.01, *** *p* < 0.001.

LEfSe analysis further confirmed distinct microbial enrichment profiles among the groups. Specifically, the Ctrl group was characterized by an enrichment of the phylum *Firmicutes* and genera from the family *Lachnospiraceae*, such as *Roseburia*, *Allobaculum*, and *Muribaculum*. In contrast, the HSD group was marked by a significant enrichment of *Clostridium sensu stricto 1* and *Ruminococcus torques group*. Notably, VTE-H treatment significantly restored the abundance of *Lachnospiraceae*, thereby remodeling the gut microbiota structure (Figure 6A,B). This suggests that VTE-H plays a key role in restoring microbial homeostasis.

Spearman correlation analysis linked microbial changes with host physiological indices. Beneficial genera such as *Lachnospiraceae*, *Roseburia*, *Bifidobacterium*, and *Dubosiella* were significantly negatively correlated with pro-inflammatory cytokines (IL-1β, TNF-α), fibrosis markers (Col1a1, α-SMA), cardiovascular dysfunction indicators (BNP, ET-1, ANF), and organ indices, but positively correlated with body weight. Conversely, harmful genera enriched under HSD, including *Clostridium sensu stricto 1* and *Ruminococcus torques group*, showed the opposite trend.

Together, these findings suggest that VTE alleviates hypertension by restoring microbial diversity and rebalancing beneficial versus pro-inflammatory taxa.

### 2.5. VTE Restores Metabolic Homeostasis in HSD-Fed Mice

**Figure 7:**
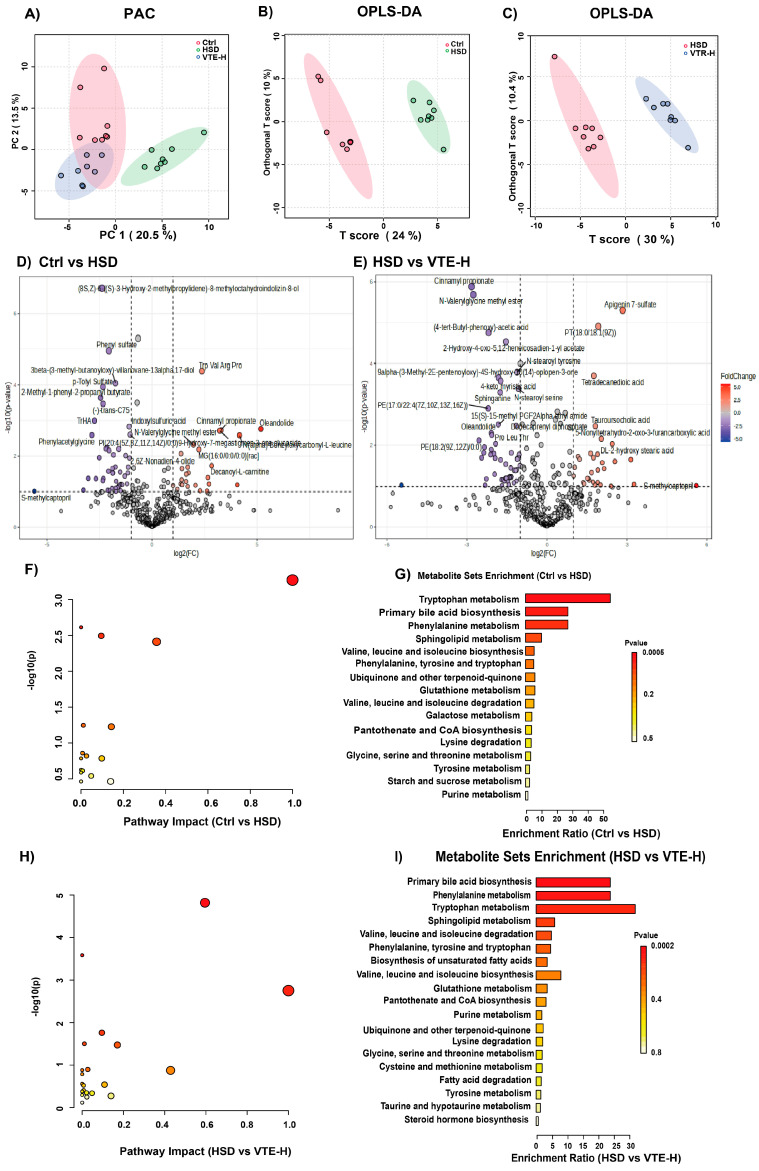
VTE ameliorates the metabolic profile in mice with HSD-induced hypertension. (**A**) Principal component analysis (PCA) score plot of metabolic profiles among the three groups. (**B**) Orthogonal projections to latent structures-discriminant analysis (OPLS-DA) score plot (Ctrl vs. HSD). (**C**) OPLS-DA score plot (HSD vs. HSD + VTE-H). (**D**) Volcano plot of differentially expressed metabolites (Ctrl vs. HSD). (**E**) Volcano plot of differentially expressed metabolites (HSD vs. HSD + VTE-H). (**F**) Pathway Impact Bubble Plot from pathway enrichment analysis (Ctrl vs. HSD). (**G**) Metabolite Sets Enrichment (Ctrl vs. HSD). (**H**) Pathway Impact Bubble Plot from pathway enrichment analysis (HSD vs. VTE-H). (**I**) Metabolite Sets Enrichment (HSD vs. VTE-H). Data are presented as mean ± SEM (*n* = 8).

Untargeted plasma metabolomics was performed to identify metabolic pathways influenced by HSD and VTE. PCA and OPLS-DA analyses revealed distinct separations among Ctrl, HSD, and HSD + VTE-H groups (Figure 7A–C). Volcano plot analysis identified 42 metabolites significantly altered in the HSD group (23 upregulated, 19 downregulated). VTE-H modified 38 metabolites (18 upregulated, 20 downregulated) (Figure 7D,E). Pathway enrichment analysis indicated that HSD predominantly affected tryptophan metabolism, primary bile acid biosynthesis, and phenylalanine metabolism (Figure 7F,G). These disturbances were largely normalized by VTE-H, which also enhanced pathways related to fatty acid metabolism, including unsaturated fatty acid biosynthesis, cysteine and methionine metabolism, fatty acid degradation, and taurine/hypotaurine metabolism (Figure 7H,I).

**Figure 8:**
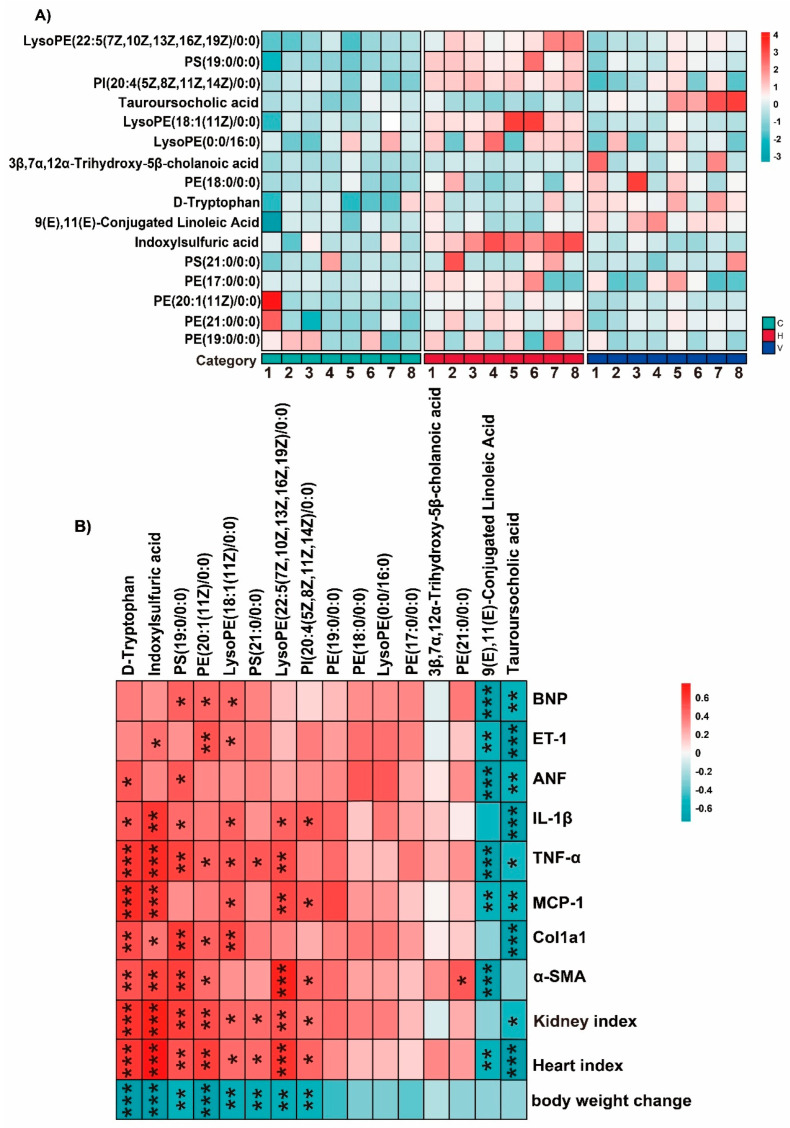
VTE improves the metabolic profile and its correlation with physiological indices in HSD-induced hypertensive mice. (**A**) Heatmap showing the relative abundance of metabolites across the three experimental groups. (**B**) Spearman’s correlation analysis between metabolites and physiological indicators (including fibrosis markers: *Col1a1*, *α-SMA*; inflammatory cytokines: *IL-1β*, *TNF-α*, *IL-6*, *IL-18*; and cardiac function parameters: *BNP*, *ET-1*, *ANF*). The color gradient represents the correlation coefficient, ranging from blue (negative correlation) to orange (positive correlation). Data are presented as mean ± SEM. * *p* < 0.05, ** *p* < 0.01, *** *p* < 0.001.

To further visualize metabolite alterations, 16 metabolites with the most significant alterations were selected for heatmap analysis. HSD feeding markedly increased indoxyl sulfate and disrupted glycerophospholipid metabolism, while reducing bile acid-related metabolites such as tauroursodeoxycholic acid (TUDCA). VTE-H treatment reversed these patterns, decreasing indoxyl sulfate levels and restoring bile acid metabolites toward control levels (Figure 8A). Correlation analysis supported these findings: indoxyl sulfate and glycerophospholipids were positively associated with inflammatory and fibrotic markers, whereas TUDCA and 9(E),11(E)-conjugated linoleic acid showed strong negative correlations with *IL-1β* expression, underscoring their anti-inflammatory potential (Figure 8B).

Collectively, these findings indicate that VTE alleviates HSD-induced metabolic dysfunction by modulating bile acid, tryptophan, and phospholipid pathways, contributing to its antihypertensive action.

### 2.6. VTE Modulates the Gut Microbiota–Metabolite Network

**Figure 9:**
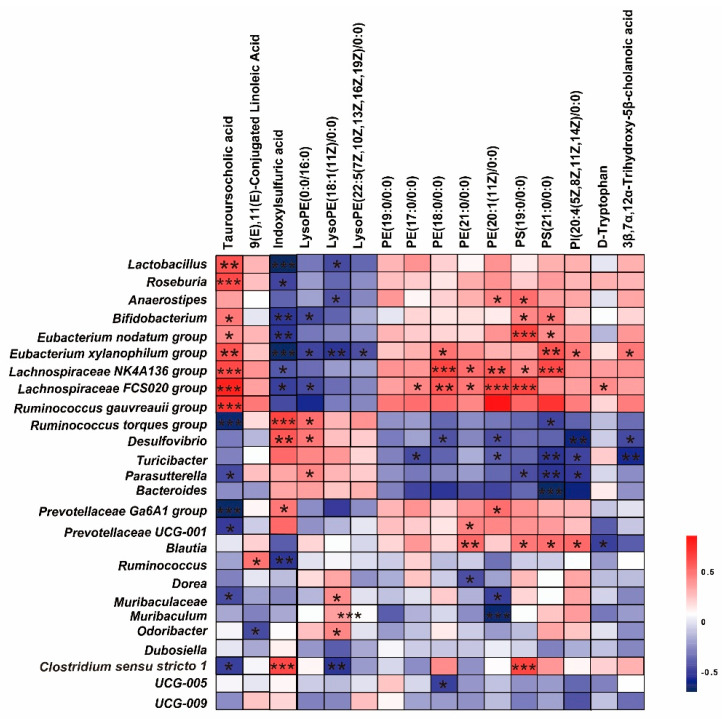
VTE modulates the correlation between gut microbiota and plasma metabolites in HSD-induced hypertensive mice. Spearman’s correlation analysis between altered gut microbial genera (genus level) and differential plasma metabolites. The color gradient represents the correlation coefficient, ranging from purple (negative correlation) to red (positive correlation). Data are presented as mean ± SEM. * *p* < 0.05, ** *p* < 0.01, *** *p* < 0.001.

To further explore the interaction between microbial and metabolic changes, correlations were analyzed between 27 gut bacterial taxa that showed significant changes and 16 differential metabolites reversed in VTE treatment. The key beneficial bacterial constituents consisted of two principal functional groups: lactic acid-producing bacteria, primarily from the genera *Bifidobacterium* and *Lactobacillus*, and butyrate-producing bacteria within the family *Lachnospiraceae*, exemplified by the genus *Roseburia*. These taxa were positively correlated with anti-inflammatory metabolites such as TUDCA, and negatively correlated with pro-inflammatory tryptophan derivatives, including indoxyl sulfate. These taxa also showed associations with glycerophospholipid-related metabolites such as LysoPE, suggesting that VTE helps restore metabolic and inflammatory balance through coordinated regulation of the gut microbiota–metabolite network (Figure 9).

### 2.7. Antibiotic Depletion Abolishes VTE’s Protective Effects

**Figure 10:**
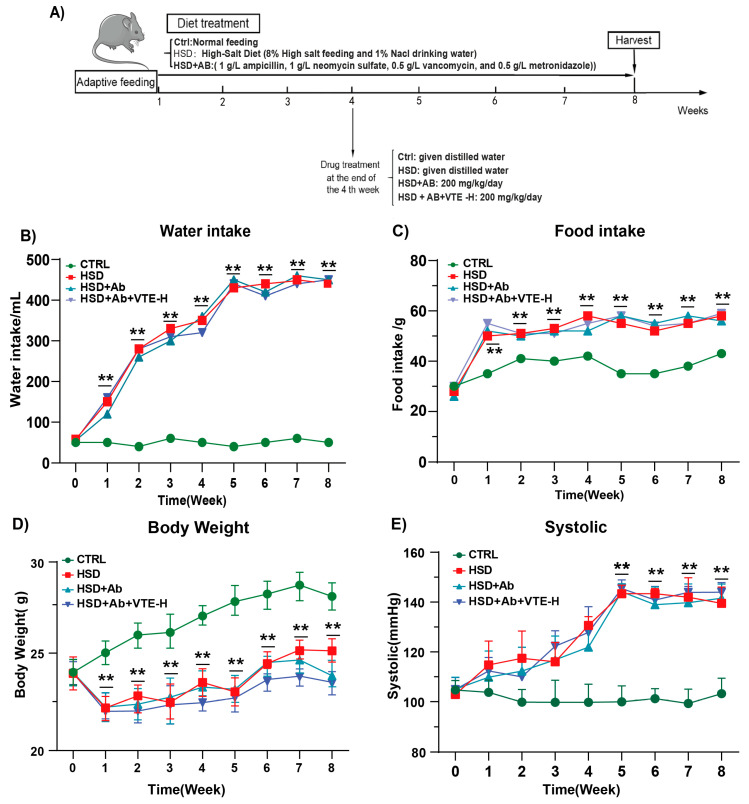
Effects of VTE on physiological parameters in antibiotic-treated mice with HSD-induced hypertension. (**A**) Schematic diagram of the experimental timeline. (**B**) Daily water intake. (**C**) Daily food intake. (**D**) Body weight changes. (**E**) Systolic blood pressure. Data are presented as mean ± SEM (*n* = 10). Data are presented as mean ± SEM (*n* = 10). ** *p* < 0.01 vs. Ctrl group.

To verify whether VTE’s antihypertensive actions require the presence of gut microbiota, mice were pretreated with a broad-spectrum antibiotic cocktail to establish pseudo-germ-free conditions (Figure 10A). Under microbiota-depleted conditions, VTE completely lost its ability to improve systemic or behavioral parameters. Food and water intake remained elevated, body weight failed to recover, and SBP was not significantly reduced compared with the HSD group (Figure 10B–E).

**Figure 11:**
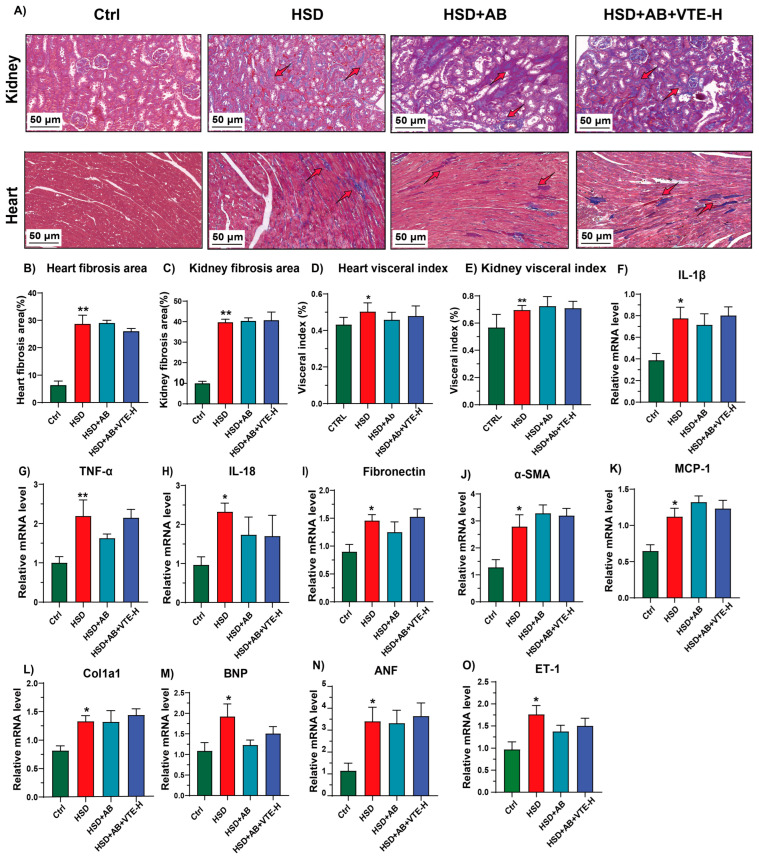
VTE fails to attenuate organ damage in microbiota-depleted mice with HSD-induced hypertension. (**A**) Representative images of heart and kidney sections stained with Masson’s trichrome (scale bar = 50 μm, magnification ×400). Red arrow indicates the area of fibrosis. (**B**) Quantitative analysis of heart fibrosis area. (**C**) Quantitative analysis of kidney fibrosis area. (**D**) Heart visceral index. (**E**) Kidney visceral index. (**F**–**O**) Relative mRNA expression levels in cardiac tissues of (**F**) *IL-1β*, (**G**) *TNF-α*, (**H**) *IL-18*, (**I**) *Fibronectin*, (**J**) *α-SMA*, (**K**) *MCP-1*, (**L**) *Col1A1*, (**M**) *BNP*, (**N**) *ANF*, (**O**) *ET-1*. Data are presented as mean ± SEM (*n* = 10). * *p* < 0.05, ** *p* < 0.01 vs. Ctrl group.

Histological examination further supported these findings. Masson staining revealed persistent structural damage in both myocardial and renal tissues of antibiotic-treated mice despite VTE treatment (Figure 11A). Masson staining and quantitative analysis showed that cardiac and renal fibrosis levels remained comparable to those in the HSD group (Figure 11B,C) and corresponding organ indices were not improved (Figure 11D,E). Consistently, qPCR analysis of cardiac tissue demonstrated that VTE treatment did not significantly alter the expression of inflammatory cytokines (*IL-1β*, *TNF-α*, *IL-18*), fibrosis-related genes (*Fibronectin*, *α-SMA*, *Col1A1*), or cardiac stress markers (*MCP-1*, *BNP*, *ANF*, *ET-1*) under microbiota-depleted conditions (*p* > 0.05 vs. HSD group) (Figure 11F–M).

Together, these results demonstrate that the antihypertensive and cardiorenal protective effects of VTE are largely dependent on the presence of gut microbiota.

## 3. Discussion

This study provides the first experimental evidence that VTE effectively alleviates HSD-induced hypertension and associated cardiorenal injury through modulation of the gut microbiota–metabolite axis. VTE treatment not only reduced SBP but also improved food and drink intake, body weight, and organ indices, accompanied by attenuation of cardiac and renal fibrosis and downregulation of inflammatory and fibrotic gene expression. These effects paralleled the restoration of gut microbial diversity and metabolic homeostasis and were abolished under antibiotic pretreatment, confirming that VTE’s protective actions are dependent on an intact gut microbiota. Collectively, these findings identify VTE as a multi-target dietary intervention that restores microbial and metabolic homeostasis, offering a safe and sustainable strategy for managing salt-sensitive hypertension.

Phytochemical analysis revealed that VTE is rich in flavonoids, including dihydromyricetin, isoquercitrin, and myricetin (Figure 1A–C), compounds well-documented for their antioxidant and anti-inflammatory properties [33,36,37,38]. Dihydromyricetin, in particular, attenuates oxidative stress, inflammation, and cardiac remodeling in cardiovascular disease models, suggesting it contributes substantially to the observed protective effects [39]. Consistent with these pharmacological properties, VTE treatment markedly improved the physiological abnormalities induced by HSD. It lowered systolic blood pressure while restoring normal food and water intake as well as body weight (Figure 2B–E). The increased food and water consumption in HSD-fed mice likely represents a compensatory response to high osmotic load. While changes in feeding behavior can indirectly affect blood pressure through metabolic, hormonal, or renal pathways [40], VTE normalized these behaviors alongside improvements in blood pressure. This temporal association suggests that behavioral recovery reflects improved systemic homeostasis rather than being the primary driver of VTE’s antihypertensive effect.

Beyond lowering blood pressure, VTE provided substantial protection against hypertensive target-organ injury. It significantly reduced cardiac and renal fibrosis and suppressed the expression of pro-inflammatory (IL-1β, TNF-α), pro-fibrotic (Col1A1, α-SMA), and cardiac stress (BNP, ET-1, ANF) markers (Figure 3 and Figure 4). Notably, although both VTE and valsartan effectively reduced SBP (Figure 2E), VTE exhibited broader protective effects against inflammation and fibrosis under these experimental conditions. Unlike valsartan, which acts mainly through AT_1_ receptor blockade [41], VTE appears to exert multi-targeted actions that restore systemic and metabolic homeostasis. These findings highlight the complementary potential of botanical interventions in hypertension management, though their clinical effectiveness requires further validation.

Consistent with its systemic effects, VTE markedly reshaped the gut microbiota disrupted by HSD, as revealed by 16S rRNA gene sequencing (Figure 5). High-salt intake induced clear dysbiosis characterized by reduced microbial richness and diversity, whereas VTE treatment restored both parameters, reestablishing ecological stability in the intestinal community. At the compositional level, VTE increased the abundance of beneficial SCFA-producing families such as *Lachnospiraceae* and *Ruminococcaceae*, which are typically depleted in hypertension [42]. These taxa are functionally significant, as SCFAs maintain endothelial integrity, regulate vascular tone, and modulate immune responses [43]. Conversely, VTE reduced several HSD-enriched pro-inflammatory genera, including *Desulfovibrio*, a sulfate-reducing bacterium that compromises barrier function and promotes systemic inflammation [44,45], and *Ruminococcus torques*, a mucin-degrading species associated with impaired barrier intestinal integrity and chronic inflammation [46,47,48]. LEfSe analysis confirmed that these bacterial groups were among the most distinct between the HSD and VTE-treated mice (Figure 6), supporting the robustness of these shifts. In contrast, valsartan produced only modest microbiota changes and failed to significantly affect *Desulfobacterota* abundance. Together, these results indicate that VTE exerts broader and more integrated effects on gut microbial composition than single-target antihypertensive drugs. This likely reflects its flavonoid-rich, multi-component nature, which promotes a balanced and resilient intestinal ecosystem that supports systemic metabolic recovery.

Untargeted plasma metabolomic profiling further revealed that VTE corrected HSD-induced metabolic disturbances, particularly in tryptophan metabolism, primary bile acid biosynthesis, and glycerophospholipid metabolism (Figure 7). These pathways are central to host–microbe communication and are closely linked to inflammation, immune homeostasis, and cardiovascular function [49,50,51]. A key finding was VTE mediated a marked reduction in indoxyl sulfate (Figure 8A); a uremic toxin derived from microbial tryptophan metabolism that induces oxidative stress, endothelial injury, and renal fibrosis [52]. Concurrently, VTE increased tauroursodeoxycholic acid (TUDCA) (Figure 8A), a bile acid that activates FXR and TGR5 signaling to suppress inflammation and strengthen intestinal barrier integrity [53]. Cross-correlation analysis showed that beneficial taxa such as *Bifidobacterium* and *Lactobacillus* were positively correlated with TUDCA and negatively correlated with indoxyl sulfate, indicating a tightly coordinated microbial–metabolic reprogramming (Figure 9). Notably, the protective effects of VTE were lost when the gut microbiota was depleted, demonstrating that its actions are microbiota-dependent (Figure 10 and Figure 11). These findings support a mechanistic model in which VTE alleviates hypertension by restoring host homeostasis via the gut microbiota–metabolite axis. This microbiota-dependent mechanism aligns with the reported actions of other plant-derived bioactives. For example, the traditional herb *Astragali Radix* ameliorates type 2 diabetes by enhancing intestinal barrier function and metabolic homeostasis via gut microbiota modulation [54], while the polyphenols cinnamic acid and cinnamaldehyde ameliorate dyslipidemia by reshaping core gut bacteria and associated metabolites [55].

Despite these promising findings, several limitations should be acknowledged. First, as a complex botanical containing multiple flavonoids, VTE’s effects likely result from synergistic interactions rather than a single active constituent. Future studies should isolate and evaluate individual components or defined fractions to identify key bioactives. Second, while antibiotic-induced microbiota depletion verified a gut-dependent mechanism, intestinal histology and barrier markers were not assessed. Third, the study was conducted in 5-week-old male C57BL/6J mice, a widely used and well-validated model for high-salt-induced hypertension and cardiovascular injure [56,57]. However, age and sex are known to influence gut microbiota composition, metabolic activity, and hypertension susceptibility. These factors may affect the translatability of the present findings, and future studies should include both sexes and a broader age range to better reflect clinical diversity. Finally, the associations between microbial and metabolic alterations remain correlative; causal relationships should be verified through targeted metabolite supplementation and receptor-specific studies

In summary, VTE mitigates HSD-induced hypertension through integrated remodeling of the gut microbiota–metabolite axis. By enriching SCFA-producing taxa and restoring key metabolic pathways, including tryptophan, bile acid, and lipid metabolism, VTE attenuates systemic inflammation and fibrosis, thereby improving vascular and renal function. This multi-target action distinguishes VTE from conventional single-pathway antihypertensive agents. These findings support vine tea as a promising, safe, and multi-target dietary adjunct for managing salt-sensitive hypertension, warranting further validation in translational and clinical studies.

## 4. Materials and Methods

### 4.1. Preparation and Compound Analysis of VTE

Based on the traditional method of consuming Chinese tea by infusion in hot water to release active compounds, vine tea extract (VTE) was prepared through hot water extraction. Vine tea (*Ampelopsis grossedentata*), commonly known as “Mei tea,” is derived from the tender stems and leaves of *A. grossedentata* (Vitaceae). Dried vine tea leaves were purchased from Laifeng Jinqi Vine Tea Biological Co., Ltd. (Wuhan, China) and authenticated by the supplier according to morphological characteristics. The dried material (0.4 kg) was ground and extracted with 4 L of water at 90 °C for 1 h. After filtration, the residue was re-extracted twice under the same conditions. All supernatants were combined and spray-dried using a Christ Alpha 1-2 LD Plus spray dryer (Martin Christ Gefriertrocknungsanlagen GmbH, Osterode, Germany). The resulting VTE powder was stored in a desiccator for further experiments. 

### 4.2. Animals and Experimental Design

Based on previous studies [56,57], 5 weeks old male C57BL/6J mice (22 ± 2 g) were selected for this study. These mice were procured from the Hubei Provincial Center for Disease Control and Prevention. All mice underwent a 1-week acclimatization period under standardized housing conditions (23 ± 2 °C, 12 h light/dark cycle) with free access to standard chow and drinking water. Following acclimatization, mice were randomly assigned to experimental groups to ensure comparable baseline characteristics.

To minimize experimental bias, this study rigorously adopted a tiered blinding approach throughout all research stages. During the group allocation phase, group assignments were concealed and only accessible to the random sequence generator, with no other researchers informed of the grouping strategy. Throughout the intervention and experimental implementation phases, the treatments administered to different groups were standardized to have identical appearance and administration procedures, enabling sustained blinding of personnel responsible for dosing and daily feeding. Additionally, to mitigate potential confounding factors, we implemented a series of systematic control strategies: all behavioral tests and histological evaluations were conducted in a randomized order; animal procurement and experimental procedures were completed in multiple independent batches, with intra-batch randomization to control for batch effects; cages from different experimental groups were arranged in a staggered “checkerboard” pattern on animal racks and rotated periodically to balance the impact of cage location. During the outcome assessment and data analysis phase, all personnel involved in behavioral scoring, histological evaluation, and statistical analysis remained fully blinded to group assignments, with access only to anonymously coded samples and datasets. The correspondence between sample codes and experimental groups was not disclosed until all statistical analyses had been completed.

In the efficacy study, mice were divided into six groups (*n* = 10/group): the control (Ctrl) group received standard diet and water; the high-salt diet (HSD) group received 8% NaCl diet and 1% NaCl drinking water; the HSD + Valsartan group received HSD and Valsartan (10 mg/kg/day, p.o.); the HSD + VTE-L, HSD + VTE-M, and HSD + VTE-H groups received HSD and low (50 mg/kg/day), medium (100 mg/kg/day), or high (200 mg/kg/day) doses of VTE, respectively. The drug intervention group started intervention 4 weeks after the initiation of HSD, and the intervention lasted for 4 consecutive weeks. Both the Ctrl group and the HSD group were administered the vehicle (sterile water) by oral gavage.

To determine whether the antihypertensive effects of VTE require the presence of gut microbiota, a separate cohort of mice was divided into four groups (*n* = 10/group): Ctrl (standard diet and water), HSD (high-salt diet), HSD + Abx (HSD with an antibiotic cocktail), and HSD + Abx + VTE (HSD with the antibiotic cocktail and VTE at 200 mg/kg/day, p.o.). The antibiotic cocktail (1.0 g/L ampicillin, 1.0 g/L neomycin, 0.5 g/L vancomycin, and 0.5 g/L metronidazole) was provided in drinking water and renewed every two days for 4 weeks. Gut microbiota depletion was confirmed by quantitative PCR showing >99% reduction in fecal bacterial 16S rRNA gene copies. No fecal microbiota transplantation or additional microbial manipulation was conducted.

Body weight and fluid/food intake were monitored daily. At the end of the treatment period, all mice were anesthetized with isoflurane and euthanized. Tissues including kidney, heart, liver, and aorta were collected, weighed for organ index calculation, and processed for subsequent histological (fixed in 4% PFA) or molecular biological (snap-frozen) analysis. All animal procedures were approved by the Animal Care and Use Committee of Hubei University of Chinese Medicine (permission number: SCXK2020-0001).

### 4.3. Blood Pressure Measurement

Body weight, along with food and water intake, was monitored and recorded daily throughout the experimental period. Systolic blood pressure (SBP) was measured in all groups at baseline (prior to the initiation of the high-salt diet) and subsequently on a weekly basis. All SBP measurements were performed non-invasively using a tail-cuff system (CODA^®^ 8-Channel High Throughput Non-Invasive Blood Pressure System, Kent Scientific, Torrington, CT, USA) by the same investigator at a consistent time of day to minimize circadian variability. Prior to measurement, mice were acclimatized in a warm chamber (maintained at ~27 °C) for 30 min to ensure sufficient vasodilation of the tail artery and stable pulse waveforms. The reported SBP values represent the mean of 15 consecutive readings obtained from each animal during a single session.

### 4.4. Histological Analysis

Heart and kidney tissues were fixed by immersion in 4% paraformaldehyde, processed through a graded ethanol series, embedded in paraffin, and sectioned at 5 μm thickness. Following deparaffinization in xylene and rehydration, tissue sections were subjected to histological staining. For morphological evaluation, sections were stained with hematoxylin and eosin (H&E) using a commercial kit (Beyotime Biotechnology, Shanghai, China). Collagen deposition was assessed using Masson’s trichrome staining kit (Zhonghui Hecai Bio-pharmaceutical Technology, Xi’an, China) according to manufacturer’s instructions.

All stained sections were examined under a Leica DMIL 4000B microscope equipped with a DFC450C digital camera (Leica Microsystems, Wetzlar, Germany). Fibrosis quantification was performed by calculating the collagen volume fraction from Masson’s trichrome-stained sections using ImageJ software v1.8.0.345 (National Institutes of Health, Bethesda, MD, USA).

### 4.5. RNA Extraction and qRT-qPCR

Total RNA was isolated from indicated tissues using Trizol reagent (Thermo Fisher Scientific, Waltham, MA, USA) according to the manufacturer’s instructions. RNA integrity was confirmed by electrophoresis. Subsequently, 500 ng of total RNA was reverse-transcribed into first-strand cDNA using the AMeasy 1st Strand cDNA Synthesis Kit (AllMEEK, Beijing, China). Quantitative real-time PCR was performed on a QuantStudio 6 Real-Time PCR System (Applied Biosystems, Waltham, MA, USA) using the 2× FastHS SYBR QPCR Mix (AllMEEK). Melting curve analysis was performed at the end of each run to confirm the specificity of amplification. The mRNA expression levels of target genes were normalized to the endogenous control β-actin. Relative gene expression was calculated using the comparative 2^−ΔΔCt^ method. Primer sequences are listed in Appendix A.2 Table A2.

### 4.6. Gut Microbiota Analysis

Fresh fecal samples were collected from individual mice at the end of treatment, snap-frozen in liquid nitrogen, and stored at −80 °C. Total genomic DNA was extracted from ~200 mg of feces using the FastDNA™ SPIN Kit (Thermo Fisher Scientific, Waltham, MA, USA) following the manufacturer’s protocol. The V3–V4 regions of the bacterial 16S rRNA gene were amplified using primers 338F (5′-ACTCCTACGGGAGGCAGCAG-3′) and 806R (5′-GGACTACHVGGGTWTCTAAT-3′), and sequenced on the Illumina MiSeq platform (2 × 250 bp, Allwegene, Beijing, China). Raw reads were processed with QIIME2 (v2023.9). After quality filtering and denoising with DADA2, amplicon sequence variants (ASVs) were clustered and taxonomically assigned against the SILVA 138 database (99% identity).

Alpha diversity indices (Chao1 richness, observed species number, PD whole tree phylogenetic diversity) were calculated to assess microbial richness and diversity; inter-group differences were analyzed using the Kruskal–Wallis’s test with Benjamini–Hochberg false discovery rate (FDR) correction. Beta diversity was evaluated using Bray–Curtis distances and visualized by principal component analysis (PCA) and partial least squares–discriminant analysis (PLS-DA); group differences were tested using permutational multivariate analysis of variance (PERMANOVA).

Taxonomic composition at phylum and family levels was summarized by relative abundance plots, and the top 50 genera were visualized in clustered heatmaps. Differentially abundant taxa were identified by Kruskal–Wallis’s test (FDR < 0.05).

LEfSe analysis was further applied to identify statistically significant and biologically relevant differential species among the four groups (LDA score threshold > 3.0). This unified threshold was consistently applied throughout the study, and multiple testing correction was incorporated within the LEfSe algorithm.

Spearman’s rank correlation analysis was performed to explore associations between key microbial genera and host parameters (e.g., systolic blood pressure, organ indices, inflammatory and fibrotic markers), with correlations visualized as heatmaps.

### 4.7. Metabolomics Analysis

Mice were anesthetized with isoflurane, and blood samples were collected via the appropriate method (e.g., retro-orbital plexus or cardiac puncture) into EDTA-K2 anticoagulant tubes. Plasma was subsequently separated by centrifugation and stored at −80 °C until analysis. Non-targeted metabolomic profiling of plasma samples was performed using an ultra-high-performance liquid chromatography-quadrupole time-of-flight mass spectrometry (UHPLC-Q-TOF/MS) system. Chromatographic separation was achieved on a ZORBAX SB-C18 column (2.1 × 100 mm, 1.8 µm) maintained at 35 °C. The mobile phase consisted of (A) 0.1% formic acid in water and (B) acetonitrile. The flow rate was 0.3 mL/min and the injection volume was 2 µL. Mass spectrometric data were acquired in both positive and negative ionization modes. Raw data were processed using MetaboAnalyst 6.0 for feature extraction, peak alignment, and normalization. Metabolite identification was conducted by matching accurate mass and MS/MS spectra against online metabolic databases (e.g., HMDB, METLIN).

### 4.8. Statistical Analysis

All statistical analyses were performed using GraphPad Prism 8.0 software (La Jolla, CA, USA). Data are expressed as the mean ± standard error of the mean (mean ± SEM). Statistical differences among groups were assessed by one-way analysis of variance (ANOVA) followed by the Tukey–Kramer post hoc test. A significance threshold of * *p* < 0.05 was applied for all statistical tests.

Spearman’s rank correlation analysis was conducted to examine the relationships between hypertension-associated bacterial genera and metabolite abundances with relevant parameters, as well as the associations between these bacterial genera and metabolites that showed significant reversal after intervention. Based on the relative abundance data of bacterial genera and metabolites across all samples, the correlation coefficient (ρ) and *p*-value were calculated for each bacterium–metabolite pair. The significance threshold was set at * *p* < 0.05, with adjustment for multiple comparisons using the false discovery rate (FDR). Results were visualized using a heatmap to illustrate significant positive and negative correlations.

## Data Availability

The data presented in this study are available on request from the corresponding author due to ethical restrictions.

## Abbreviations

The following abbreviations are used in this manuscript:

Ab	Antibiotic
ANP	Atrial natriuretic peptide
α-SMA	Alpha smooth muscle actin
BNP	Brain natriuretic peptide
Col1A1	Collagen type I alpha 1 chain
ET-1	Endothelin-1
HSD	High-salt diet
H&E	Hematoxylin-eosin staining
IL-1β	Interleukin-1β
IL-6	Interleukin-6
IL-18	Interleukin-18
MCP-1	Monocyte chemotactic protein-1
RT-qPCR	Quantitative reverse transcription PCR
SCFAs	short-chain fatty acids
SBP	Systolic blood pressure
TNF-α	Tumor necrosis factor-α
TUDCA	Tauroursodeoxycholic acid
VTE	Vine tea Extract
VTE-H	High-dose VTE treatment

## Appendix A

### Appendix A.1

**Table A1:** Identified major chemical compounds in VTE.

Peak	Retention Time (min)	Molecular Formula	Molecular Weight	[M+H]^+^/[MH] (*m*/*z*)	MS/MS Fragments (*m*/*z*)	Identification
1	1.991	C_7_H_6_O_5_	170.12	169.0141	125.0245, 97.0294, 79.0190(10)	Gallic acid
2	3.987	C_15_H_14_O_7_	306.272	307.0818	139.0390, 2889.0706, 181.0499(10)	(+)-Gallocatechin
3	5.097	C_27_H_30_O_17_	626.1468	625.1552	409.0565, 391.0440, 371.0950(40)	Quercetin 3-O-gentiobioside
4	8.004	C_36_H_28_O_16_	716.1363	761.1345	745.6231, 726.0345, 699.7159(10)	Theaflavin-3′-gallate
5	8.887	C_15_H_14_O_6_	290.0793	291.0865	139.0390, 123.0442, 273.07699(10)	(−)-Epicatechin
6	8.934	C_14_H_12_O_4_	244.0738	289.0721	245.0816, 205.0816, 109.0297(10)	Piceatannol
7	9.635	C_14_H_20_O_7_	319.0479	323.1103	315.5350, 294.1756, 279.4797(10)	Salidroside
8	10.446	C_20_H_20_O_11_	436.1008	481.0992	355.0667, 193.0143, 463.0771(10)	Swertianolin
9	10.826	C_15_H_12_O_8_	320.250	319.0463	301.0353, 192.0142, 125.0243(20)	Dihydromyricetin
10	11.080	C_17_H_24_O_9_	372.1426	395.13	233.0781(10), 364.1127, 233.0780, 185.0419(20)	Syringin
11	12.041	C_22_H_18_O_10_	442.379	441.0816	331.0447, 169.0147, 305.0669(10)	(−)-Catechin gallate(−)-
12	12.774	C_15_H_14_O_6_	290.273	291.09	139.0387, 123.0440, 165.0544(10)	(+)-Catechin
13	15.147	C_21_H_20_O_13_	480.382	479.083	317.0293(10), 359.041, 341.0491(20)	Myricetin 3-O-β-D-glucopyranoside
14	15.895	C_15_H_10_O_6_	286.241	285.0403	269.0449, 241.0496, 217.0499(10)	Kaempferol
15	16.028	C_27_H_30_O_16_	610.526	607.0715	589.0614, 571.0518, 545.0710(10)	Kaempferol 3-O-sophoroside
16	16.263	C_15_H_12_O_7_	304.0586	305.0659	179.0338, 287.0551, 153.0175(10)	Taxifolin
17	16.661	C_21_H_20_O_10_	432.384	437.1444	438.2391, 439.2426(20), 438.2390(40)	Trilobatin
18	17.293	C_21_H_20_O_13_	480.0908	481.098	91.0389, 319.0449(10), 319.0450(20)	Myricetin 3-O-galactoside
19	17.423	C_21_H_18_O_13_	478.0743	477.067	316.0223(10), 287.0228, 271.0240(20)	Quercetin 3-O-β-D-glucuronide
20	17.708	C_21_H_22_O_10_	434.1213	457.1103	127.0373, 185.0401, 377.0283(10)	Naringenin-7-O-β-D-glucoside
21	17.874	C_22_H_28_O_10_	452.1663	453.1737	307.1155(20), 321.0479, 275.1564(40)	4′-O-β-D-glucosyl-5-O-methylvisamminol
22	18.871	C_15_H_10_O_8_	318.0381	319.0452	153.0183(10), 273.0379, 245.0449 (20)	Myricetin
23	19.051	C_21_H_20_O_12_	464.0955	463.0884	316.022(10), 287.0196, 271.0243(20)	Isoquercitrin
24	21.211	C_20_H_18_O_11_	434.085	433.0777	300.0270, 273.0769, 167.0350(20)	Avicularin
25	21.545	C_21_H_20_O_11_	448.383	471.0898	185.0413, 310.0369, 428.4682(10)	Homoorientin l
26	22.340	C_15_H_10_O_7_	302.0432	303.0504	301.0352(10), 301.0346, 271.0243(20)	Quercetin
27	22.343	C_21_H_20_O_11_	448.1012	471.0906	325.0320, 169.0470(10), 325.0321(20)	Kaempferol 7-O-β-D-glucoside
28	24.104	C_22_H_30_O_6_	390.2047	413.1941	119.0861, 232.0670(10), 294.1430(20)	Pregomisin
29	24.134	C_21_H_20_O_10_	432.1055	431.0982	285.0403, 255.0287(10), 227.0344(20)	Aloe-emodin-3-(hydroxymethyl)-O-β-D-glucopyranoside
30	25.615	C_21_H_18_O_12_	462.0804	463.0883	444.7671, 427.0651, 359.0382(10)	Kaempferol-3-O-β-D-glucuronide
31	29.187	C_21_H_36_O_10_	448.2311	471.2204	527.8887, 435.6232, 510.8288(10)	Atractyloside A

### Appendix A.2

**Table A2:** Primer Sequence Table.

Gene	Forward (5′-3′)	Reverse (5′-3′)
β-Actin	CGTGAAAAGATGACOCAGA	GTCCATCACAATGCCTGT
IL-1β	GGCTGGACTGTTTCTAATGC	ATGGTTTCTTGTGACCCTGA
TNF-α	GGGTGTTCATCCATTCTC	GGAAAGCCCATTTGAGT
IL-6	GAAACCGCTATGAAGTTCCTCTCT	TGTTGGGAGTGGTATCCTCTGTGA
IL-18	GCAGCAGGTGGAATTGTATCG	TGTGCCTCCCCAGAGGATT
Fibronectin	ACGAAGTCAGTGTCTATGC	GAAGCCAGTGATTGTCTC
α-SMA	TGGCTATTCAGGCTGTGCTGTC	CAATCTCAOGCTCGGCAGTA
MCP-1	CAATAGGAAGATCTCAGTGCAGAGG	GGAATOCTGAAOCCACTTCT
Col1A1	AGGTCGGTGTGAACGGATTTG	TGTAGACCATGTAGTTGAGGTCA
BNP	AAGTCCTAGCCAGTCTCCAGAACAA	AAACAACCTCAGCCCGTCACAG
ANF	TTGGAGCCCAGAGTGGACTA	ACACACCACAAGGGCTTAGG
ET-1	CTCTTCTTGCCGGTTGGGAA	TTTCTACAGAAACCCCGCCC
